# *Chlamydia trachomatis*’ struggle to keep its host alive

**DOI:** 10.15698/mic2017.03.564

**Published:** 2017-03-02

**Authors:** Barbara S. Sixt, Raphael H. Valdivia, Guido Kroemer

**Affiliations:** 1 INSERM U1138, Centre de Recherche des Cordeliers, Paris 75006, France.; 2 Equipe 11 labellisée par la Ligue Nationale Contre le Cancer, Centre de Recherche des Cordeliers, Paris 75006, France.; 3 Université Paris Descartes, Paris 75006, France.; 4 Metabolomics and Cell Biology Platforms, Institut Gustave Roussy, Villejuif 94800, France.; 5 Department of Molecular Genetics and Microbiology, Duke University, Durham, NC 27710, USA.; 6 Pôle de Biologie, Hôpital Européen Georges-Pompidou, AP-HP, Paris 75015, France.; 7 Karolinska Institute, Department of Women's and Children's Health, Karolinska University Hospital, Stockholm 17176, Sweden.

**Keywords:** Chlamydia, vacuolar pathogen, inclusion membrane protein, cell death, interferon response, cell-autonomous defense, virulence

## Abstract

Bacteria of the phylum *Chlamydiae *infect a diverse range of eukaryotic host species, including vertebrate animals, invertebrates, and even protozoa. Characteristics shared by all *Chlamydiae *include their obligate intracellular lifestyle and a biphasic developmental cycle. The infectious form, the elementary body (EB), invades a host cell and differentiates into the replicative form, the reticulate body (RB), which proliferates within a membrane-bound compartment, the inclusion. After several rounds of division, RBs retro-differentiate into EBs that are then released to infect neighboring cells. The consequence of this obligatory transition between replicative and infectious forms inside cells is that *Chlamydiae *absolutely depend on the viability and functionality of their host cell throughout the entire infection cycle. We recently conducted a forward genetic screen in *Chlamydia trachomatis*, a common sexually transmitted human pathogen, and identified a mutant that caused premature death in the majority of infected host cells. We employed emerging genetic tools in *Chlamydia *to link this cytotoxicity to the loss of the protein CpoS (*Chlamydia *promoter of survival) that normally localizes to the membrane of the pathogen-containing vacuole. CpoS-deficient bacteria also induced an exaggerated type-1 interferon response in infected cells, produced reduced numbers of infectious EBs in cell culture, and were cleared faster from the mouse genital tract in a transcervical infection model *in vivo*. The analysis of this CpoS-deficient mutant yielded unique insights into the nature of cell-autonomous defense responses against *Chlamydia *and highlighted the importance of *Chlamydia*-mediated control of host cell fate for the success of the pathogen.

The capacity of *Chlamydia *spp. to block the apoptotic machinery in infected cells is well documented. Obviously, it is tempting to speculate that the premature loss of the replicative niche represents a potential threat that *Chlamydia *actively counteracts to ensure its survival and replication in eukaryotic hosts. The nature of this threat may be multifaceted (Fig. 1). *Chlamydia*-infected cells are exposed to potentially lethal signals originating from immune cells, such as cytotoxic lymphocytes (including NK- or T-cells) and death-inducing cytokines. But pro-death signals may also arise from within the infected cell, for example as a consequence of infection-induced metabolic stress, disruption of host cellular functions, or DNA damage. *Chlamydia *spp. can block apoptosis induced by both immune mediators (such as granzyme B or TNF-α) and insults that trigger intracellular stress (such as irradiation, staurosporine, or etoposide). A third possible source of pro-death stimulation may reside in the cell-intrinsic induction of programmed host cell death pathways in response to the engagement of intracellular innate immune sensors. Such a response has been described in leukocytes like macrophages, but is less well explored in epithelial cells, which represent the major host target for human-adapted *Chlamydia *pathogens.

**Figure 1:**
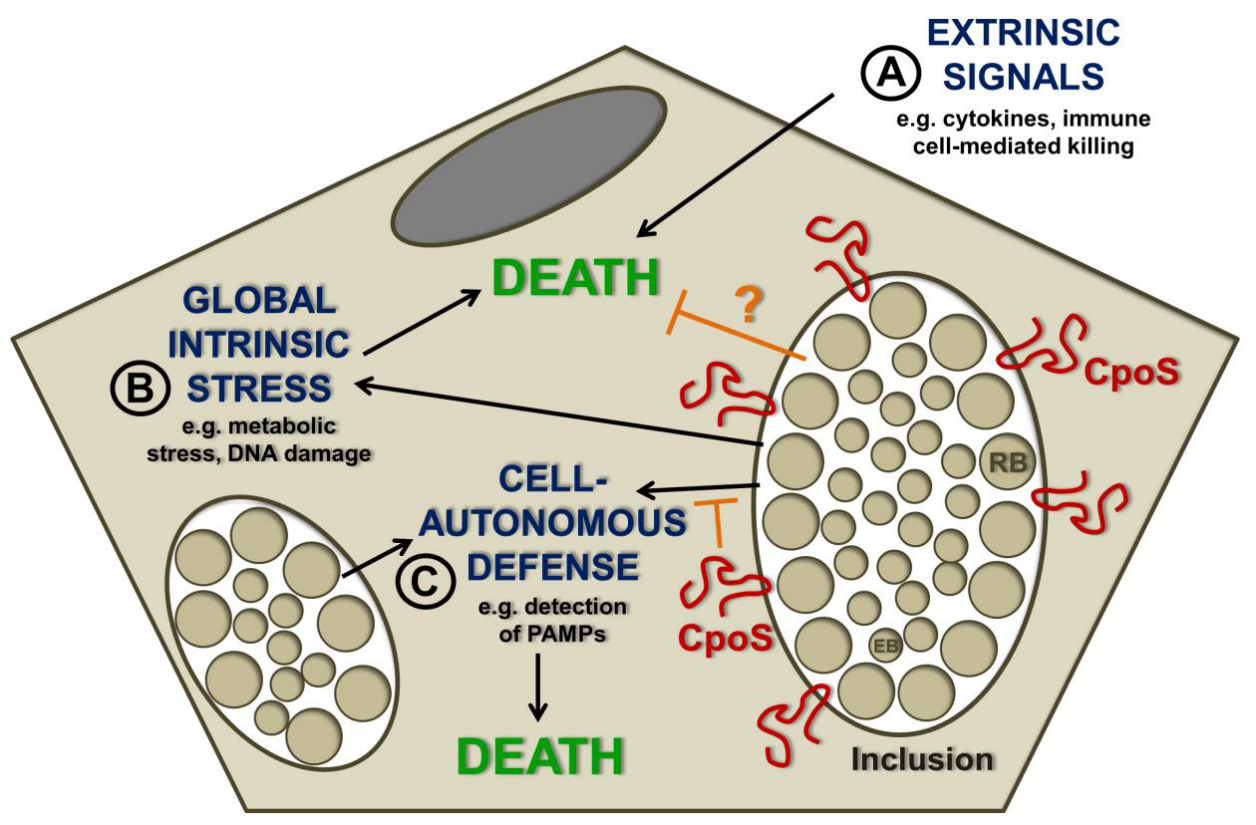
FIGURE 1: Possible sources of pro-death signals acting on *Chlamydia*-infected cells. **(A)** Inside the host, *Chlamydia*-infected cells are exposed to cytotoxic cytokines and death-inducing immune cells, but have evolved to block induction of cell death by these stimuli. **(B)** The ability of *Chlamydia *spp. to block apoptosis induced by cellular stress inducers suggests that the bacteria actually need to protect host cells from infection-induced lethal stress. **(C)** Cell death may be induced by activation of immune sensors that regulate cell-autonomous defense mechanisms. Our recent study demonstrated that the *C. trachomatis *inclusion membrane protein CpoS counteracts the activation of the PAMP sensor STING and prevents emission of pro-death signals from the inclusion.

During a screen for *C. trachomatis *mutants that triggered spontaneous premature host cell death, we identified a strain that lacked a key protein (CpoS) on the membrane of the inclusion. CpoS-defective bacteria induced early host cell death in numerous cell lines, including epithelial cells. Multiple complementary experiments led us to the conclusion that CpoS-defective bacteria fail to protect their host from strong pro-cell death signals that emanate as a result of the host recognizing the inclusion. Under most circumstances, when cells are co-infected with two different *Chlamydia *strains, the inclusions they form fuse to create a single large inclusion. This is a process mediated by the inclusion membrane protein IncA. When epithelial cells were co-infected with a CpoS- and a CpoS+ strain, their inclusions fused, CpoS was expressed on the surface of the hybrid inclusion, and the cell was protected from death. In contrast, when a cell was co-infected with a CpoS- mutant and a CpoS+ IncA- strain (which abrogated the fusion among inclusions) the cell was no longer protected from death. These findings demonstrated that CpoS acts in a spatially restricted manner to block pro-death signals that arise from within the inclusion in which it resides (Fig. 1).

The molecular events that lead to the demise of the host cell in response to infection with CpoS-deficient bacteria are not completely understood. In cell culture, cytotoxicity was diminished by inhibitors of host transcription or translation, although protection was only observed when the drugs were added early after infection. These findings suggested that the lethal response depended on the *de novo *synthesis of host factors. It is also clear that cell death is at least partially dependent on the engagement of programmed cell death signaling pathways, because a proportion of dying cells displayed typical features of apoptosis, including membrane blebbing and activation of apoptotic effector caspases. Other cells succumbed to necrosis, i.e. a sudden rupture of the plasma membrane that was not preceded by an apoptotic morphology. Future experimentation must examine the potential contribution of programmed necrosis pathways, albeit neither pyroptosis-deficient cells nor cells treated with necroptosis inhibitors appeared to be significantly protected.

The transcriptional response of host cells infected with CpoS-deficient *C. trachomatis *indicated a strong induction of genes encoding cytokines, including TNF-α and type-1 interferons (IFNs), as well as an increased expression of IFN-stimulated genes. This enhanced IFN response was dependent on the cGAS/STING/TBK1/IRF3 pathway, which transduces the detection of pathogenic cytosolic DNA or bacterial cyclic-di-nucleotides into interferon expression. We observed that the immune sensor protein STING was more robustly activated in cells infected with CpoS-deficient *Chlamydia*, suggesting enhanced sensing of the pathogen by the host cell. While this enhanced awareness of infection can also contribute to the induction of host cell death, we excluded that toxicity is a direct consequence of increased cytokine responses. Time-lapse microscopic observations indicated that inclusion-free cells adjacent to infected cells, including both genuinely uninfected cells as well as inclusion-free cells that arose from infected cells by division, did not undergo cell death, although these cells were exposed to the same cytokine environment. Moreover, pharmacologic or genetic inhibition of the IFN pathway failed to protect cells from death. However, genetic ablation of STING caused a partial protection from the overmortality of host cells infected by CpoS-deficient *Chlamydia*, suggesting that STING may regulate cell survival independently of its role in IFN signaling. Nevertheless, significant cell death induction by CpoS-deficient bacteria was also observed in cells like HEK293T cells that do not express STING, demonstrating that STING cannot be the sole factor mediating cell death induction by CpoS-deficient bacteria.

Because STING activation depends on its translocation to post-endoplasmic reticulum (ER) vesicles, it is possible that CpoS counteracts this process by modulating vesicular trafficking via its ability to recruit host Rab GTPases to the inclusion. Alternatively, CpoS may modulate the availability or accessibility of chlamydial pathogen-associated molecular patterns (PAMPs) for detection by host receptors, for example by conferring structural stability to the inclusion membrane. For some vacuolar bacterial pathogens it is known that enhanced leakage of bacterial components into the cytosol can induce type-1 IFN responses and cell death, at least in macrophages. However, within the limits of fluorescence microscopy and time-lapse analyses of live cells infected with CpoS-deficient *Chlamydia*, we did not observe extravacuolar bacterial growth. Time-lapse video microscopy also indicated that in cells undergoing necrotic death the rupture of the inclusion membrane occurred either simultaneously with the rupture of the host cell membrane or immediately before (Fig. 2). In some apoptotic cells inclusion integrity appeared to be preserved throughout the membrane blebbing stage. These observations suggested that the induction of the IFN and cell death responses by CpoS-deficient bacteria do not depend on premature, cataclysmic rupture of the inclusion. Nonetheless, further experiments are required to achieve the proper temporal resolution of the events that precede cell death including more sensitive assays to detect subtle changes in inclusion membrane integrity and PAMP accessibility.

**Figure 2:**
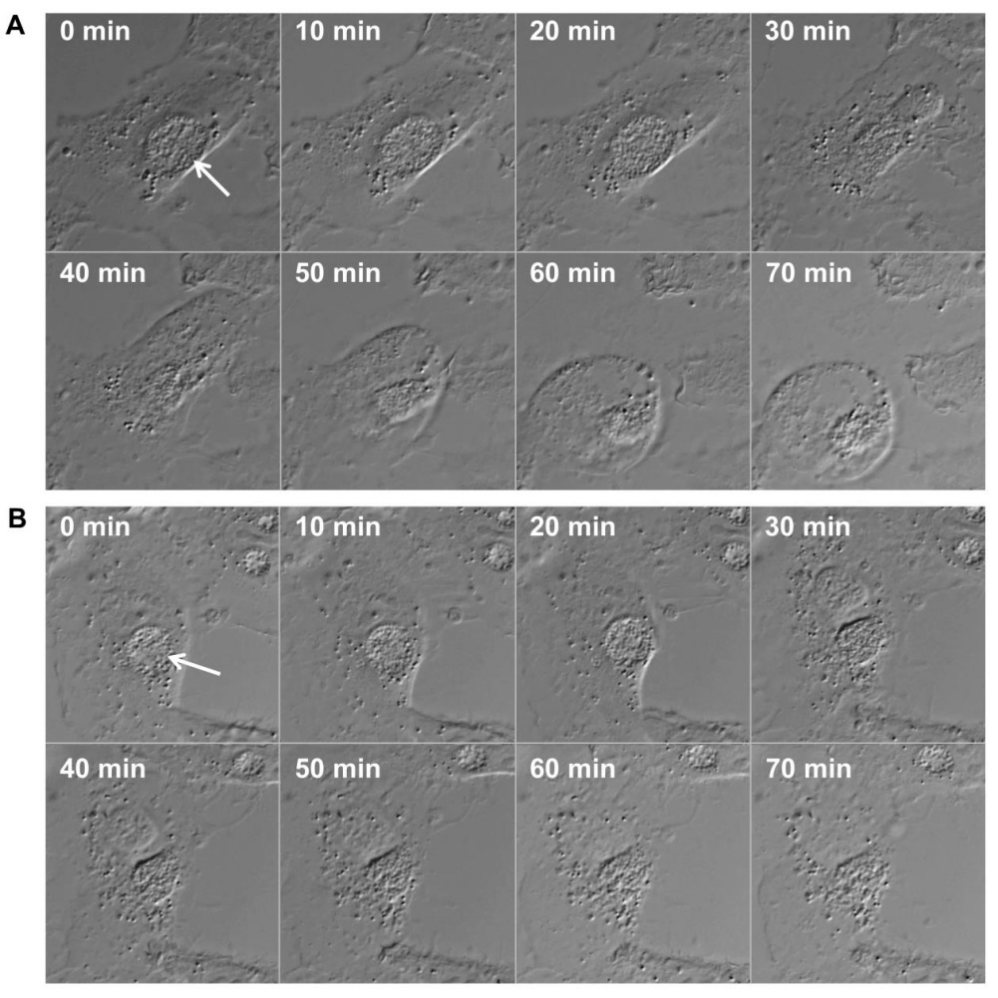
FIGURE 2: Time lapse microscopic image series displaying inclusion and plasma membrane rupture in HeLa cells infected with CpoS-deficient *C. trachomatis*. HeLa cells (ATCC CCL-2) were infected with CpoS-deficient *C. trachomatis *L2/434/Bu (CTL2-*cpoS*::*bla, *10 inclusion-forming units/cell) and imaged for over 40 hours in 10-min intervals at an Axio Observer.Z1 microscope (Zeiss). The displayed image series **(A and B)** document the process of necrotic cell death in two selected cells. The morphologic changes in these dying cells suggest that the rupture of the inclusion membrane occurred simultaneously to, or immediately before, the rupture of the plasma membrane. Arrows indicate inclusions.

Experiments in cell culture indicated that CpoS-deficient *C. trachomatis *strains produce significantly lower numbers of infectious EBs than their CpoS-proficient counterparts. This could be a synergistic outcome of the enhanced production of type-1 IFNs, which may restrict *Chlamydia *replication in infected cells, and the premature loss of the replicative niche. We initially envisioned that, in the animal host, exaggerated cell death and cytokine responses could also result in enhanced tissue damage and pathology. However, infections of mice indicated that CpoS-deficient bacteria are cleared faster from tissues leading to a diminished cytokine response in the murine genital tract. Interestingly, CpoS is among the most polymorphic inclusion membrane proteins in *C. trachomatis*, and CpoS homologs are only found in two other *Chlamydia *species, *C. muridarum *and *C. suis*, which are natural pathogens of mice and swine, respectively. We do not know whether CpoS function is dispensable during infection with other *Chlamydia *species or whether its functions can be taken over by other effector proteins. Moreover, it remains to be determined whether subtle changes in CpoS structure/function among distinct *Chlamydia *species may influence their host or tissue tropism. We predict that further elucidation of the mode of action of CpoS and related *Chlamydia *effectors will not only greatly enhance our understanding of *Chlamydia *virulence, but will also reveal new facets of host cell signaling pathways that mediate cell-autonomous defense responses to infectious agents.

